# Polymer-Based Scale Inhibition and Desorption Behavior in Carbonate Reservoirs: Core Flooding Investigation and Statistical Modeling

**DOI:** 10.3390/polym18111336

**Published:** 2026-05-28

**Authors:** Soroush Ahmadi, Azizollah Khormali

**Affiliations:** 1 Department of Chemical Engineering, Faculty of Petroleum, Gas, and Petrochemical Engineering, Persian Gulf University, Bushehr P.O. Box 7516913817, Iran; 2 Department of Chemistry, Faculty of Basic Sciences and Engineering, Gonbad Kavous University, Gonbad Kavous P.O. Box 4971799151, Iran

**Keywords:** polyphosphinocarboxylic acid, polymeric scale inhibitor, desorption, statistical modeling, squeeze treatment, scale inhibition efficiency

## Abstract

Scale deposition, particularly calcium sulfate, poses a major challenge in carbonate reservoirs, leading to permeability reduction and operational inefficiencies. In this study, the performance of a polymeric scale inhibitor, polyphosphinocarboxylic acid (PPCA), was systematically investigated through dynamic core flooding experiments combined with statistical modeling. To address scale inhibition performance and minimum inhibitor requirements, additional static jar tests and dynamic tube blocking experiments were conducted. The results confirmed a minimum inhibitory concentration (MIC) of 40 ppm PPCA, where inhibition efficiency exceeded 90% at elevated temperatures. Moreover, the desorption behavior of PPCA was evaluated under a wide range of operational conditions, including pore volume (0–40 PV), temperature (50–100 °C), injection rate (2–6 mL/min), and pH (6–8). Effluent concentrations were quantified using a spectrophotometric method and expressed as the C_f_/C_i_ ratio (effluent concentration to injected concentration) to characterize inhibitor return behavior. A comprehensive dataset comprising 224 experimental runs was analyzed using Response Surface Methodology (RSM), leading to the development of two predictive models for low (0–10 PV) and high (10–40 PV) pore volume ranges. The models demonstrated excellent predictive capability, with R^2^ values of 0.9934 and 0.9979, respectively. In addition, statistical analysis confirmed that pore volume and injection rate were the most influential parameters, while pH exhibited a comparatively minor effect. Results showed that increasing PV, temperature, injection rate, and pH led to a decrease in C_f_/C_i_, indicating enhanced desorption. For instance, C_f_/C_i_ decreased from approximately 0.12 at 10 PV to 0.06 at 40 PV under reference conditions. Furthermore, optimization results revealed that maintaining an effective inhibitor concentration (C_f_/C_i_ more than 0.05) is strongly dependent on operating conditions. At 60 °C, a wide operational window was observed, whereas at 100 °C, the effective region significantly narrowed due to accelerated desorption. Furthermore, permeability reduction analysis (K_d_/K_i_) demonstrated significant suppression of scale-induced formation damage in the presence of PPCA, while blank tests showed severe permeability decline. The integrated results validate the dual role of PPCA in both scale inhibition efficiency and formation protection under dynamic conditions. The novelty of this work lies in integrating polymer-specific behavior with dynamic core flooding and multivariable statistical modeling, providing a robust predictive framework for optimizing squeeze treatment design in carbonate reservoirs.

## 1. Introduction

Mineral scale deposition is a persistent and costly challenge in oil and gas production systems, particularly in carbonate reservoirs where geochemical conditions favor precipitation reactions [1,2]. Among the various inorganic scales, calcium sulfate (CaSO_4_) is one of the most problematic due to its low solubility, high thermal stability, and strong tendency to adhere to rock surfaces and production equipment. Scale formation typically occurs when incompatible waters mix, such as formation brines rich in calcium ions (Ca^2+^) and injected seawater containing sulfate ions (SO_4_^2−^) [3,4]. This leads to supersaturation, nucleation, and subsequent crystal growth within the porous media and production tubing. The consequences include pore throat blockage, permeability reduction, increased pressure drop, and ultimately significant declines in hydrocarbon production. In severe cases, scale deposition can also lead to equipment failure and costly shutdowns, emphasizing the need for effective scale control strategies [5,6,7].

Chemical scale inhibition is widely regarded as one of the most efficient and economically viable methods for mitigating scale formation [8,9,10,11,12,13]. Among various inhibitor classes, polymer-based scale inhibitors have attracted considerable attention due to their versatility, tunable chemical structure, and strong interaction with both ions in solution and mineral surfaces. These polymers typically function through multiple mechanisms, including threshold inhibition, crystal distortion, dispersion, and adsorption. Their performance is largely governed by molecular characteristics such as functional group type, molecular weight, charge density, and chain conformation. In particular, polymers containing phosphonate and carboxylate functional groups have demonstrated superior performance in inhibiting calcium-based scales due to their strong chelation ability and adsorption affinity [14,15,16].

Polyphosphinocarboxylic acid is a highly effective polymeric scale inhibitor that combines phosphonate and carboxylic functional groups within its molecular structure. From a polymer chemistry perspective, PPCA can be considered a multifunctional copolymer with both anionic charge density and flexible backbone architecture, allowing it to interact efficiently with cationic species such as Ca^2+^ [17,18]. The presence of phosphonate groups enhances its binding strength with metal ions, while carboxylate groups contribute to solubility and dispersion capabilities. This dual functionality enables PPCA to inhibit scale formation through both chelation and adsorption mechanisms. Additionally, its polymeric nature allows for multiple binding sites along the chain, increasing its effectiveness even at low concentrations. These characteristics make PPCA particularly suitable for challenging environments such as high-temperature and high-salinity carbonate reservoirs [19,20,21].

In carbonate formations, scale inhibitors are commonly applied using squeeze treatments, where the inhibitor solution is injected into the reservoir and allowed to adsorb onto the rock surface. The retained inhibitor is gradually released over time, providing prolonged protection against scale formation during production [22,23]. The success of such treatments depends on the adsorption-desorption behavior of the inhibitor, which is influenced by both reservoir conditions and polymer properties [24,25]. From a polymer science viewpoint, adsorption is controlled by electrostatic interactions, hydrogen bonding, and surface complexation between functional groups of the polymer and the carbonate rock surface. Desorption, on the other hand, is governed by flow dynamics, ionic strength, and competitive ion interactions [20,26,27]. Therefore, understanding the interplay between polymer structure and reservoir conditions is critical for optimizing inhibitor performance.

Experimental evaluation of scale inhibitors has traditionally relied on static methods such as jar tests, which provide initial insights into inhibition efficiency under controlled conditions [15]. However, these methods do not adequately capture the dynamic nature of fluid flow and transport processes occurring in porous media. Core flooding experiments offer a more representative approach by simulating reservoir conditions, including fluid flow, pressure gradients, and rock-fluid interactions [28,29,30,31]. In these experiments, inhibitor solutions are injected through core samples, and parameters such as pressure drop, effluent concentration, and permeability changes are monitored. This allows for the assessment of inhibitor placement, retention, and release behavior, as well as its effectiveness in preventing scale formation under realistic conditions [32,33,34,35].

The behavior of polymeric inhibitors such as PPCA in core flooding experiments is influenced by several operational parameters. Pore volume injection determines the extent of inhibitor placement within the core and affects adsorption distribution. Temperature plays a crucial role in both reaction kinetics and polymer stability, influencing adsorption strength and desorption rates [36,37,38]. Injection rate affects the residence time of the inhibitor within the porous medium and can alter transport mechanisms such as advection and dispersion [39,40]. pH is another critical factor, as it directly impacts the ionization state of functional groups in the polymer, thereby affecting its charge density and interaction with both ions and rock surfaces [41,42]. These parameters are often interdependent, making it challenging to evaluate their combined effects using conventional experimental approaches.

PPCA stability under a wide range of temperatures and salinities enhances their applicability in diverse reservoir conditions [43]. However, its performance can still be affected by factors such as competitive adsorption with other ions, mechanical degradation under high shear rates, and potential interactions with other chemical additives. These challenges highlight the need for a systematic and integrated approach to evaluating and optimizing polymer performance [23,44].

To address the complexity of such multivariable systems, statistical modeling techniques have been increasingly utilized. RSM is a powerful tool for designing experiments, developing empirical models, and optimizing processes involving multiple variables. RSM uses regression analysis to establish relationships between independent variables and response parameters, while also capturing interaction effects [45,46]. In the context of polymer-based scale inhibition, RSM can be used to model key responses such as inhibition efficiency, return concentration, and permeability reduction. This approach not only reduces the number of required experiments but also provides valuable insights into the relative importance of different factors and their interactions. The integration of experimental core flooding data with RSM modeling provides a comprehensive framework for understanding and optimizing the performance of polymeric scale inhibitors [47]. By systematically varying parameters such as pore volume injection, temperature, injection rate, and pH, it is possible to generate a robust dataset that captures the dynamic behavior of PPCA under realistic conditions. The application of RSM to this dataset enables the development of predictive models that can be used to identify optimal operating conditions and improve the design of squeeze treatments. Furthermore, such models can serve as valuable tools for scaling laboratory results to field applications, reducing uncertainty and improving operational efficiency [48,49].

Extensive research has been conducted on the behavior of polymeric scale inhibitors, particularly PPCA, in carbonate formations with emphasis on their adsorption, precipitation, and overall performance in squeeze treatments. Previous mechanistic studies by Jarrahian and Sorbie demonstrated that the retention of scale inhibitors such as PPCA in carbonate rocks is governed by a coupled adsorption/precipitation mechanism, where precipitation becomes dominant at higher concentrations and temperatures [50]. Their findings also highlighted the strong dependence of inhibitor retention on pH and temperature, with PPCA showing higher retention at lower pH values. Investigations by authors into the role of mineralogy revealed that both calcite and dolomite significantly influence inhibitor behavior, with precipitation of SI-Ca complexes being the dominant retention mechanism beyond certain concentration thresholds [51]. These studies emphasized that carbonate minerals exhibit higher chemical reactivity compared to sandstone, leading to more complex retention behavior. Research by Farooqui and Sorbie demonstrated that the solubility and inhibition efficiency of PPCA are strongly dependent on its molecular weight fractions, especially in precipitation squeeze treatments [11]. In addition, core flooding studies by Andrei and Malandrino have shown that precipitation squeeze treatments of PPCA can significantly enhance squeeze lifetime compared to conventional adsorption methods, emphasizing the importance of selecting appropriate operational conditions to promote controlled precipitation within the formation [52]. Recent advancements by Ghorbani et al. for PPCA have also explored innovative approaches to enhance adsorption capacity, such as the use of nanomaterials, where significantly higher adsorption levels have been reported compared to conventional rock systems, although such methods remain largely at the laboratory scale and require further validation [17]. From a modeling perspective, statistical techniques such as RSM have been widely applied in petroleum engineering to analyze and optimize complex multivariable processes. Previous studies, including those by Khormali and Ahmadi have demonstrated the capability of RSM to accurately predict scale precipitation behavior under dynamic conditions, while other works have confirmed its effectiveness in optimizing enhanced oil recovery and upgrading processes [53]. These studies highlight the robustness and importance of RSM as a predictive and optimization tool in petroleum engineering applications.

Despite the recognized effectiveness of PPCA as a polymeric scale inhibitor, existing studies have largely focused on either static evaluation methods or limited dynamic experiments, without fully capturing the complex interactions between operational parameters and polymer behavior in porous media. In particular, there is a lack of comprehensive investigations that integrate polymer science concepts, such as functional group ionization, chain conformation, and adsorption mechanisms, with dynamic core flooding data under varying pore volume injection, temperature, injection rate, and pH conditions. Moreover, the combined effects and interactions of these parameters have not been systematically modeled using advanced statistical approaches, limiting the ability to accurately predict and optimize inhibitor performance. Therefore, the primary aim of this study is to experimentally evaluate the performance of PPCA in carbonate core samples through core flooding experiments under a wide range of operational conditions, and to develop a predictive and optimization framework using RSM. The novelty of this work lies in the integration of polymer-specific behavior with dynamic core flooding analysis and multivariable statistical modeling, enabling a deeper understanding of adsorption-desorption mechanisms and their dependence on physicochemical conditions. This approach provides a more realistic and comprehensive evaluation of PPCA performance, while also offering a robust predictive tool for optimizing squeeze treatment design and enhancing calcium sulfate scale inhibition in carbonate reservoirs.

## 2. Materials and Methods

### 2.1. Materials

The scale inhibitor used in this study was phosphino-polycarboxylic acid (PPCA), supplied as a commercial aqueous formulation under the trade name Bellasol^®^ CA, manufactured by Italmatch (Naples, Italy), product grade: industrial oilfield scale-inhibitor grade, product code/catalog designation: BLS-PPCA-42. The material was supplied as an aqueous solution containing 42.2 wt% active polymer, and was used as received without further purification. According to the manufacturer’s technical data sheet and certificate of analysis, the active polymer purity exceeded 95 wt%, with the remaining balance consisting primarily of deionized water and trace stabilizing additives (<5 wt%). The product had a reported density of 1.21 ± 0.02 g cm^−3^ (25 °C) and pH of 2.8–3.5 in the supplied form.

**Figure 1:**
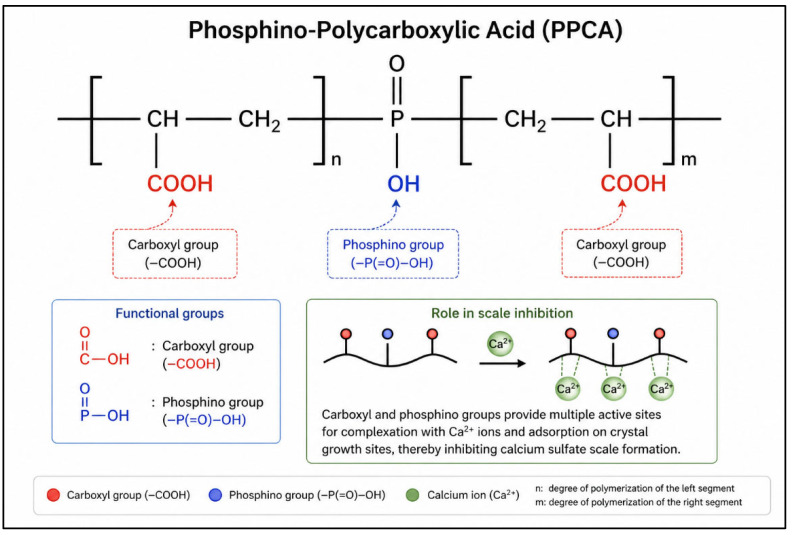
Schematic illustration of the PPCA molecular structure showing the arrangement and distribution of phosphino and carboxylate functional groups along the polymer backbone [54].

The average molecular weight of PPCA was reported by the manufacturer to be in the low-to-medium range (Mn = 1000–5000 g mol^−1^), with a polydispersity index of approximately 1.8, indicating moderate molecular weight distribution suitable for reservoir transport and adsorption applications. Structurally, the polymer contains both phosphonate and carboxylate functional groups distributed along the polymer backbone, with a phosphonate-to-carboxylate molar ratio of approximately 1:2.3. These functional groups provide multiple active coordination sites for Ca^2+^ complexation and adsorption onto carbonate mineral surfaces. The overall anionic charge density, reported by the supplier, was 4.8 ± 0.3 meq g^−1^ at pH 7, confirming the strong polyelectrolyte character of the material. These physicochemical characteristics are consistent with the expected adsorption–desorption behavior of PPCA in carbonate porous media and provide the necessary basis for interpreting the experimental core-flooding results. Structurally as shown in Figure 1, PPCA contains both phosphonate and carboxylate functional groups distributed along its polymer backbone, resulting in high anionic charge density and multiple active binding sites. These chemical characteristics play a critical role in the inhibition process by promoting strong complexation with calcium ions and adsorption onto mineral surfaces, thereby enhancing scale control performance under dynamic flow conditions. A stock solution was prepared using deionized water and diluted to the required concentration (40 ppm) for all core flooding experiments.

Two types of brines were prepared to represent formation water and injection water. The formation water was designed to be rich in calcium ions, while the injection water contained a higher concentration of sulfate ions to promote calcium sulfate scale formation under mixing conditions as shown in Table 1. Analytical-grade salts, including calcium chloride (CaCl_2_, purity > 99%) and sodium sulfate (Na_2_SO_4_, purity > 99%), were dissolved in deionized water to prepare the brines. The ionic composition and salinity of both solutions were selected based on typical reservoir conditions reported for carbonate formations. All solutions were filtered through 0.45 µm membrane filters to remove suspended particles and ensure consistency during injection.

Carbonate core samples used in this study were obtained from Iranian reservoirs, representing typical carbonate lithology encountered in oilfield operations. The cores consisted predominantly of limestone and dolomite, which are the main lithological components of many carbonate reservoirs.

The mineralogical composition of the carbonate core samples was determined using a conventional carbonate characterization approach based on acid digestion analysis, supported by petrographic thin-section observations and supplier-provided routine core analysis data. Initially, representative powdered samples obtained from the core plugs were subjected to selective acid dissolution using standardized hydrochloric acid procedures to quantify total carbonate content. The relative proportions of calcite and dolomite were then estimated based on differential acid reactivity and stoichiometric carbonate balance. Non-carbonate fractions, including quartz and clay minerals, were identified and semi-quantified using petrographic thin-section microscopy. This combined methodology is commonly used for routine reservoir-core mineralogical characterization when advanced diffraction-based methods are unavailable. To ensure reliability, measurements were conducted in triplicate, and the average values are reported with an estimated analytical uncertainty of ±2 wt%.

The results (Table 2) indicated that the core samples were composed primarily of calcite (68 wt%) and dolomite (27 wt%), with minor amounts of quartz (3 wt%) and clay minerals (2 wt%). The high carbonate content confirms the representative nature of the selected samples for typical carbonate reservoirs. The presence of both calcite and dolomite is particularly important, as these minerals exhibit different surface charge characteristics and reactivity toward polymeric scale inhibitors, which may influence the adsorption and desorption behavior of PPCA during core flooding experiments. The average porosity of the core samples was approximately 19%, and the permeability was around 27 mD, indicating a moderately porous and permeable formation. Prior to experimentation, the core samples were cleaned using standard Soxhlet extraction with toluene and methanol to remove any residual hydrocarbons and impurities. The cleaned cores were then dried in an oven at 60 °C until a constant weight was achieved. Core dimensions, including length and diameter, were measured precisely, and petrophysical properties such as porosity and permeability were confirmed using conventional laboratory methods. These properties are essential for interpreting flow behavior and inhibitor transport during core flooding experiments.

Deionized water was used throughout the study for solution preparation and system cleaning. All reagents used were of analytical grade to ensure experimental accuracy and reproducibility.

### 2.2. Static Jar and Dynamic Tube Blocking Tests

Static jar tests were conducted to evaluate the calcium sulfate scale inhibition efficiency of PPCA under controlled batch conditions. Synthetic formation water containing calcium ions and synthetic injection water containing sulfate ions were prepared separately using analytical-grade salts dissolved in deionized water. The ionic compositions were selected to simulate representative carbonate reservoir brines. The two brines were mixed at a 50:50 volumetric ratio, creating supersaturated conditions favorable for calcium sulfate precipitation.

PPCA was added at concentrations of 10, 20, 30, 40, and 50 ppm, and experiments were conducted at 50, 75, and 100 °C under constant pH = 7. After inhibitor addition and brine mixing, samples were aged under static conditions for 24 h in sealed glass containers placed in a temperature-controlled oven to ensure complete precipitation equilibrium. Following aging, the solutions were filtered, and the remaining dissolved calcium concentration in the supernatant was measured using a standard EDTA titration method (calcium ion analysis), following established water-analysis protocols.

The inhibition efficiency (IE, %) was calculated using:

(1) $$IE=\frac{{\left[{Ca}^{2+}\right]}_{3}-{\left[{Ca}^{2+}\right]}_{2}}{{\left[{Ca}^{2+}\right]}_{1}-{\left[{Ca}^{2+}\right]}_{2}}$$

where ${\left[{Ca}^{2+}\right]}_{3}$ is the residual calcium concentration in the inhibited sample, ${\left[{Ca}^{2+}\right]}_{2}$ is the residual calcium concentration in the blank (without inhibitor), and ${\left[{Ca}^{2+}\right]}_{1}$ is the initial calcium concentration before precipitation. All experiments were performed in duplicate, and the reported values represent average results. Experimental uncertainty was estimated to be ±3%.

Dynamic tube blocking tests were performed to determine the dynamic minimum inhibitor concentration (MIC) and evaluate PPCA performance under flowing conditions. The apparatus consisted of a 1 m stainless-steel capillary tube with an internal diameter of 1 mm, connected to two high-precision injection pumps supplying formation water and injection water separately. The two brines were mixed continuously at a 50:50 volumetric ratio immediately before entering the capillary tube to induce calcium sulfate supersaturation under dynamic conditions.

Experiments were conducted at constant pH = 7, while PPCA concentrations of 0, 10, 20, 30, and 40 ppm were tested. The pressure drop across the tube was continuously monitored during 120 min of injection. In the absence of sufficient inhibition, scale deposition inside the tube caused progressive blockage and a sharp pressure increase. The MIC was defined as the minimum PPCA concentration at which no significant pressure increase occurred during the test period.

All experiments were performed in duplicate, and the uncertainty of pressure measurements was estimated as ±5 kPa. The dynamic tube blocking method provides direct evaluation of inhibitor performance under realistic transport and precipitation conditions, complementing the static jar-test results.

### 2.3. Core Flooding Tests

**Figure 2:**
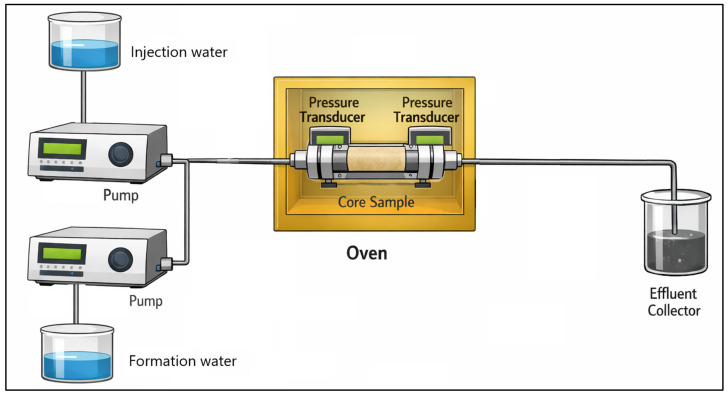
The schematic of the used apparatus for coreflood experiments.

Core flooding experiments were conducted to evaluate the adsorption and desorption behavior of PPCA under dynamic conditions that simulate fluid flow in carbonate reservoirs. The experimental setup consisted of a core holder, high-pressure injection pumps, fluid accumulators, a back-pressure regulator, and an oven to control temperature. The core holder was designed to maintain confining pressure around the core sample to prevent bypass flow and to ensure that fluid flow occurred exclusively through the porous medium. The schematic of the used apparatus is shown in Figure 2.

Prior to each experiment, the core sample was vacuum-saturated with formation water to ensure complete saturation and to remove any trapped air. The saturated core was then mounted inside the core holder, and a confining pressure of 1500 psi was applied to ensure proper sealing and to prevent fluid bypass around the core sample during flooding experiments. In addition, a back-pressure of 500 psi was maintained throughout all experiments to simulate reservoir pressure conditions, ensure single-phase flow, and avoid gas evolution during high-temperature flooding. The system was then brought to the desired experimental temperature using a temperature-controlled oven, and sufficient time was allowed to reach thermal equilibrium.

The core flooding procedure consisted of two main stages: adsorption (squeeze stage) and desorption (production stage).

(I) During the adsorption stage, a PPCA solution with a concentration of 40 ppm was injected into the core samples. The purpose of this stage was to allow the polymeric inhibitor to adsorb onto the carbonate rock surface through electrostatic interactions, hydrogen bonding, and surface complexation mechanisms. The injected volume was controlled in terms of pore volumes (PV), ensuring consistent placement of the inhibitor within the porous medium.
(II) Following the adsorption stage, the system was switched to the desorption stage, during which inhibitor-free brine was injected through the core. This process simulates production conditions, where the retained inhibitor gradually desorbs and is transported out of the formation. The desorption process was monitored over a range of 0 to 40 PV, providing a comprehensive evaluation of inhibitor return behavior and retention capacity.

A key variable investigated in this study was pH, which plays a critical role in controlling the ionization state of PPCA functional groups and, consequently, its adsorption behavior. Three pH levels (6, 7, and 8) were examined. A calibrated pH meter was used to measure and verify the pH before injection. To maintain stable pH conditions during experiments, buffer solutions were avoided to prevent interference with ionic interactions; instead, pH was monitored periodically, and fresh solutions were prepared as needed to ensure consistency. Moreover, the injection rate was varied at three levels (2, 4, and 6 mL/min) to investigate its effect on inhibitor transport and retention. Lower injection rates provide longer residence time for the polymer within the porous medium, potentially enhancing adsorption, while higher injection rates promote faster transport and reduced interaction time. Temperature was also varied at three levels (50, 75, and 100 °C) to evaluate its influence on polymer stability, adsorption strength, and desorption kinetics.

During the desorption stage, effluent samples were collected at regular intervals corresponding to specific pore volumes. The concentration of PPCA in the effluent was measured up to 40 PV and expressed as the ratio of effluent concentration to injected concentration (C_f_/C_i_). This dimensionless parameter provides a clear indication of inhibitor return behavior and retention efficiency. Monitoring C_f_/C_i_ as a function of injected pore volume allows for the construction of desorption curves, which are essential for understanding inhibitor release dynamics and predicting field performance. It should be noted that the concentration of PPCA in the effluent samples was determined using a spectrophotometric method. This method is based on the conversion of phosphonate groups present in PPCA into orthophosphate through chemical digestion, followed by reaction with ammonium molybdate and a reducing agent to form a blue-colored phosphomolybdenum complex. The intensity of the developed color was measured using a UV-Vis spectrophotometer at an appropriate wavelength, and the absorbance was correlated with PPCA concentration. A calibration curve was prepared using standard PPCA solutions with known concentrations to ensure accurate and reliable quantification.

All measurements were conducted at least in duplicate to ensure reproducibility, and the average values were reported. The experimental dataset obtained from these core flooding tests, including C_f_/C_i_ profiles up to 40 PV, was subsequently utilized for modeling and optimization using RSM, as described in the subsequent sections.

### 2.4. Statistical RSM Methodology

In this study, four input variables were considered (Table 3): pore volume, designated as PV (A), ranging from 0 to 40; temperature (B), evaluated at three levels of 50, 75, and 100 °C (−1, 0, and +1); injection rate (C), at three levels of 2, 4, and 6 mL/min (−1, 0, and +1); and pH (D), at three levels of 6, 7, and 8 (−1, 0, and +1).

In this investigation, 224 experimental runs were performed to provide comprehensive coverage of the design space and to accurately capture the system’s nonlinear response. Each run represents a distinct combination of operating conditions, as listed in Table A1. The measured response for all experiments was the C_f_/C_i_ ratio. The laboratory results were subsequently analyzed using RSM, expressed through the following general equation:

Y = β_0_ + ∑βiXi + ∑βiiXi^2^ + ∑∑βijXiXj         (i, j = 1, 2, 3 … k) (2)

where Y denotes the response, Xi refers to the ith independent variable, β_0_ is the intercept, βi and βii correspond to the linear and quadratic coefficients, and βij represents the interaction coefficient between any two input variables.

## 3. Results and Discussion

### 3.1. Static and Dynamic Evaluation of Scale Inhibition Performance and Determination of Minimum Inhibitor Concentration

To further support the scale inhibition behavior and to validate the practical effectiveness of PPCA, static jar and dynamic tube blocking tests were conducted to evaluate inhibition efficiency and determine the minimum inhibitor concentration (MIC) under representative operating conditions.

Static scale inhibition experiments were performed according to a conventional jar test protocol under constant pH = 7, using PPCA concentrations ranging from 10 to 50 ppm, and temperatures of 50, 75, and 100 °C. In each test, calcium-containing and sulfate-containing brines were mixed in the presence of the inhibitor and aged under controlled temperature conditions. After the reaction period, the remaining dissolved calcium concentration was measured, and inhibition efficiency (IE%) was calculated.

**Figure 3:**
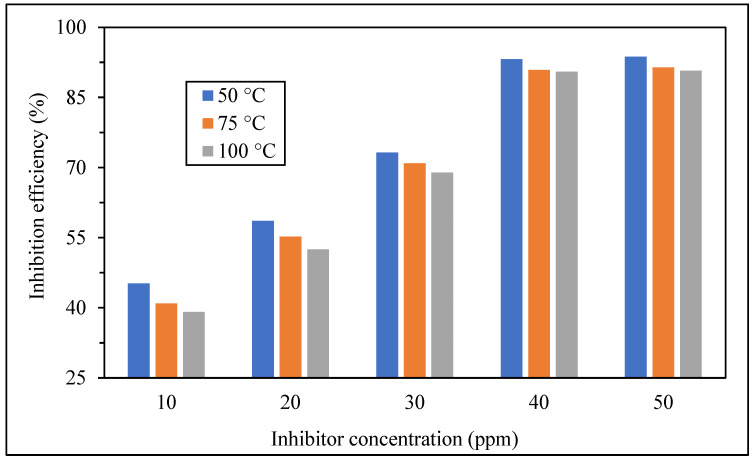
Static jar test results for PPCA inhibition efficiency.

As shown in Figure 3, inhibition efficiency increased systematically with increasing PPCA concentration at all temperatures. At 50 °C, IE increased from 45.2% (10 ppm) to 93.7% (50 ppm); at 75 °C, it increased from 40.9% to 91.4%; and at 100 °C, from 39.1% to 90.7%. A slight reduction in performance at elevated temperature was observed, which is attributed to accelerated nucleation kinetics and increased scale precipitation tendency. Importantly, at 40 ppm, inhibition efficiency exceeded 90% under all tested temperatures (93.2%, 90.9%, and 90.5%, respectively), indicating that this concentration is sufficient to provide effective static scale control.

To confirm inhibitor performance under flowing conditions, dynamic tube blocking experiments were conducted using a stainless-steel capillary tube (1 m length, 1 mm internal diameter) under continuous flow conditions. Incompatible brines were injected simultaneously to induce calcium sulfate precipitation, while PPCA concentration was varied from 0 to 40 ppm. Pressure drop across the tube was continuously monitored for 120 min, and inhibitor performance was evaluated based on the ability to suppress pressure buildup caused by scale deposition.

**Figure 4:**
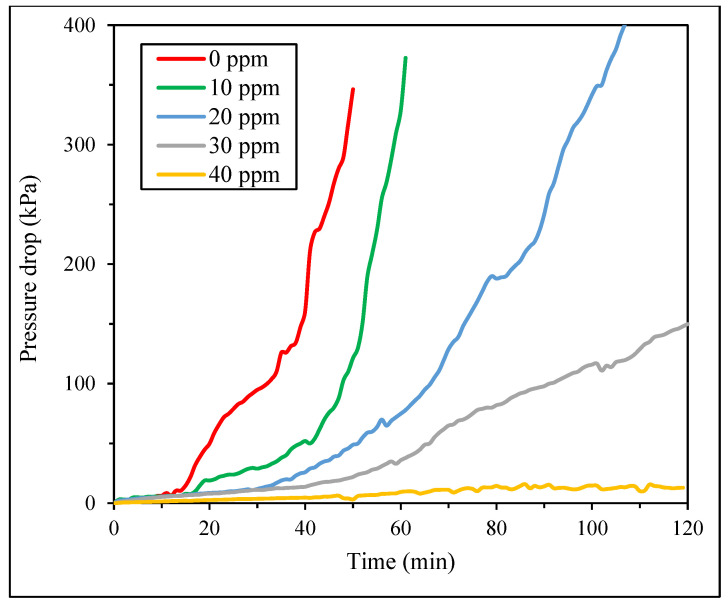
Dynamic tube blocking results showing PPCA MIC for calcium sulfate scale inhibition.

As shown in Figure 4, in the blank test (0 ppm) and at 10, 20, and 30 ppm, rapid pressure increase was observed, reaching values greater than 400 kPa, confirming severe tube blockage due to scale formation. In contrast, at 40 ppm, the pressure remained nearly constant throughout the entire 120 min test and did not exceed 15 kPa, demonstrating complete suppression of scale deposition under dynamic conditions. This confirms that 40 ppm represents the MIC for effective calcium sulfate inhibition in the studied system.

Overall, the combined static and dynamic results strongly support the selection of 40 ppm PPCA used in the coreflooding experiments and confirm that the desorption behavior analyzed in this work is directly linked to an inhibitor concentration capable of delivering effective field-relevant scale control.

### 3.2. Model Development

RSM is a widely applied technique for modeling and analyzing complex systems. An RSM-based model provides a mathematical description of the relationship between the response variable and the independent input variables, enabling optimization of experimental conditions and reliable prediction of system behavior [55,56,57,58]. In this study, the experimental data presented in Table A1 were analyzed using RSM through ANOVA, leading to the development of two predictive models, **C_f_/C_i_-PV010** and **C_f_/C_i_-PV1040**, to estimate the ratio of effluent concentration to injected concentration (C_f_/C_i_). The formulated RSM models are summarized in Table 4. Specifically, the C_f_/C_i_-PV010 model was designed to predict C_f_/C_i_ within the low-to-moderate PV range (0–10), whereas the C_f_/C_i_-PV1040 model applies to the moderate-to-high PV range (10–40).

The ANOVA results for the developed models are provided in Table 5 and Table 6. ANOVA serves as a statistical method to evaluate the overall significance of the models as well as the individual model terms, including main effects and interaction effects, based on their corresponding *p*-values and F-values. In this work, model adequacy and parameter significance were assessed at a 95% confidence level. Accordingly, a model or model term is considered statistically significant when the *p*-value is less than or equal to 0.05 and the F-value is sufficiently large [59,60,61,62,63].

**Figure 5:**
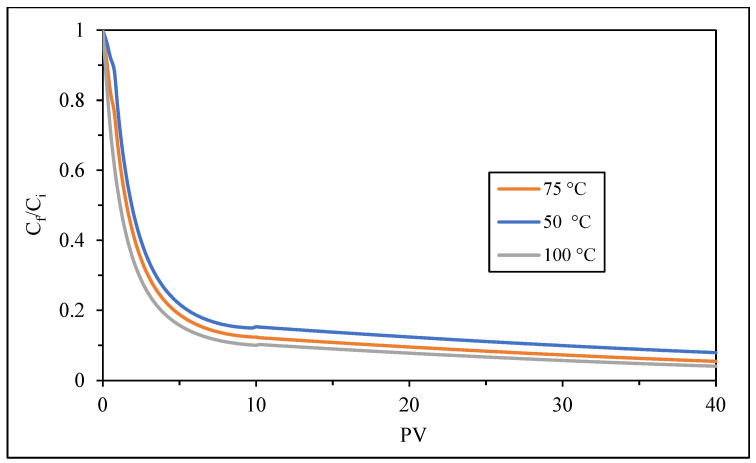
Predicted PPCA squeeze lifetime (C_f_/C_i_) up to 40 PV at different temperatures.

To further evaluate the practical validity period of the PPCA squeeze treatment, the developed RSM models were used to predict the long-term desorption profile (C_f_/C_i_) up to 40 PV under fixed operational conditions of pH = 7 and injection rate = 4 mL/min at three temperatures (50, 75, and 100 °C), as shown in Figure 5. The predicted curves demonstrate a typical exponential decline in inhibitor return concentration with increasing injected pore volume, which is characteristic of controlled desorption from carbonate surfaces. At all temperatures, the highest inhibitor return was observed at early PV, followed by a rapid decline during the initial desorption stage and a slower gradual decrease at later PV, indicating progressive depletion of the adsorbed polymer layer.

Temperature showed a pronounced effect on squeeze lifetime. At 50 °C, the inhibitor exhibited the slowest desorption rate, with C_f_/C_i_ remaining at approximately 0.080 after 40 PV, indicating prolonged inhibitor retention and the longest effective squeeze duration. At 75 °C, the final C_f_/C_i_ decreased to about 0.055, reflecting moderate thermal acceleration of desorption. In contrast, at 100 °C, the inhibitor concentration declined much faster, reaching approximately 0.041 at 40 PV, confirming significant reduction in squeeze lifetime under harsher thermal conditions. This behavior is attributed to weakening of hydrogen bonding and surface-complexation interactions between PPCA functional groups and carbonate active sites, combined with increased polymer-chain mobility at elevated temperature. These results clearly demonstrate that temperature is a key controlling factor for the operational lifetime of PPCA squeeze treatments and should be carefully considered in field design to ensure sustained scale protection.

As shown in Table 5 and Table 6, the *p*-values for both the C_f_/C_i_-PV010 and C_f_/C_i_-PV1040 models were below 0.0001 (far less than 0.05), confirming that the developed models are statistically significant. It is also important to note that there is only a 0.01% probability that the very high F-values obtained for C_f_/C_i_-PV010 (1513.73) and C_f_/C_i_-PV1040 (4396.03) could occur due to random noise, demonstrating that these models are highly significant. Furthermore, model terms with *p*-values below 0.05 are regarded as significant, whereas those with *p*-values above 0.1 are considered insignificant. According to Table 5, the significant terms in the C_f_/C_i_-PV010 model include: PV (A), temperature (B), injection rate (C), pH (D), the interaction terms AB, AC, AD, and the quadratic effects A^2^, B^2^, and C^2^. For the C_f_/C_i_-PV1040 model, Table 6 shows that A, B, C, D, AB, AC, AD, A^2^, B^2^, C^2^, and D^2^ are all significant. The statistical results also reveal that PV, followed by injection rate, exerted the greatest influence on C_f_/C_i_ in both models, as reflected by their comparatively large F-values, whereas pH had the smallest effect among the primary parameters.

### 3.3. Model Fit Summary

In this study, the R^2^, adjusted R^2^ (Adj-R^2^), predicted R^2^ (Pred-R^2^), and adequate precision (AP) were used as key statistical indicators to assess how well the model fits the experimental data. An R^2^ value approaching 1 indicates strong agreement between the model predictions and the actual measured values. In general, a robust and reliable model is characterized by high R^2^ statistics that are also closely aligned with one another, signifying good consistency between experimental observations and predicted outcomes [64,65,66]. In addition, adequate precision was employed to assess the signal-to-noise (S/N) ratio, where a value greater than 4 is considered acceptable and indicative of a sufficient model signal. The fit statistics for the developed C_f_/C_i_-PV010 and C_f_/C_i_-PV1040 models are summarized in Table 7.

As shown in this table, the C_f_/C_i_-PV010 model yielded an R^2^ value of 0.9934, indicating that the model accounts for 99.34% of the variability in the response. The adjusted R^2^ and predicted R^2^ were calculated as 99.27% and 99.15%, respectively, demonstrating strong agreement between them (their difference is less than 0.2%) and confirming the high significance of the model. For the C_f_/C_i_-PV1040 model, the R^2^ statistics were determined to be 99.79% (R^2^), 99.77% (Adj-R^2^), and 99.72% (Pred-R^2^), as presented in Table 7. These high values further support the strong significance and reliability of the model.

Additionally, the Adequate Precision values for the C_f_/C_i_-PV010 and C_f_/C_i_-PV1040 models were 151.3442 and 270.1669, respectively. These results clearly show that the AP values far exceed the threshold value of 4, confirming that both models possess sufficient signal strength and are suitable for navigating the design space. Meanwhile, the coefficients of variation (C.V.) for the C_f_/C_i_-PV010 and C_f_/C_i_-PV1040 models were 2.40% and 1.63%, respectively. Such low C.V. values indicate minimal variability relative to the mean, reflecting high experimental reliability and confirming the development of a precise and robust response model.

### 3.4. Validation of the Model Using Diagnostic Analyses

One of the primary methods used to evaluate the validity and adequacy of a developed model is the analysis of diagnostic plots. These plots help determine whether the selected experimental design and fitted model are capable of accurately representing and approximating the actual experimental observations.

**Figure 6:**
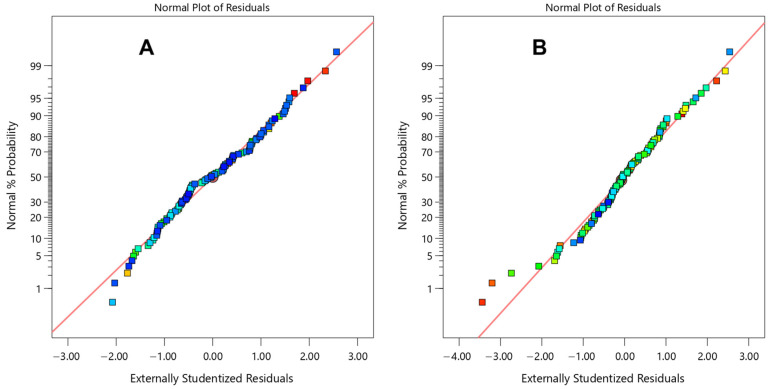
Normal probability plot of studentized residuals for C_f_/C_i_-PV010 (**A**) and C_f_/C_i_-PV1040 (**B**).

**Figure 7:**
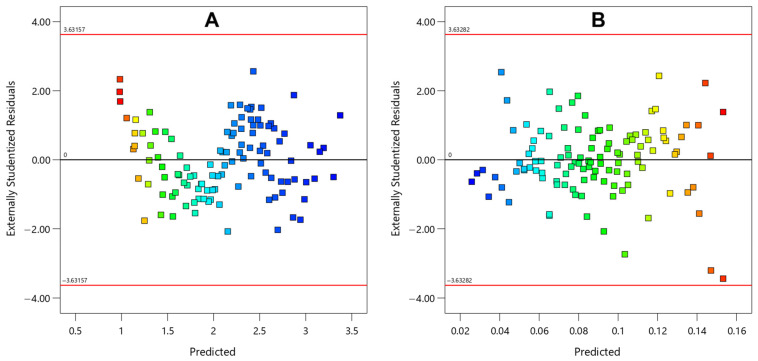
Plot of residuals versus predicted values for C_f_/C_i_-PV010 (**A**) and C_f_/C_i_-PV1040 (**B**).

**Figure 8:**
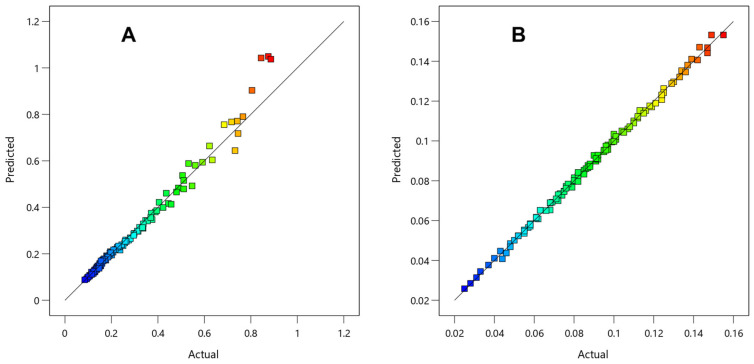
Plot of predicted values versus actual measurements for C_f_/C_i_-PV010 (**A**) and C_f_/C_i_-PV1040 (**B**).

Figure 6 presents the normal probability plot of externally studentized residuals, which is commonly used to assess whether the residuals follow a normal distribution. For a well-fitted model, the data points should lie approximately along a straight line. As illustrated in this figure, the residuals of the developed models generally align with the diagonal reference line, indicating that the assumption of normal distribution is satisfied. The residuals versus predicted values for the C_f_/C_i_ models are shown in Figure 7. This type of plot is used to evaluate whether the residuals are randomly distributed and whether they exhibit constant variance. In an appropriate model, the points should be randomly scattered around zero without any visible trend or systematic pattern. As observed in the figure, the residuals are randomly distributed with no identifiable structure and show approximately constant variance, further confirming the adequacy of the developed model. In addition, Figure 8 illustrates the relationship between the predicted values and the corresponding experimental results for C_f_/C_i_. The data points are closely distributed around the diagonal line, demonstrating strong agreement between the experimentally measured values and those predicted by the model. Therefore, the diagnostic plot analysis clearly verifies the validity of the Cf/Ci-PV010 and Cf/Ci-PV1040 models and confirms that the proposed model is sufficiently reliable for predicting the ratio of effluent concentration to injected concentration.

### 3.5. Sensitivity Analysis

**Figure 9:**
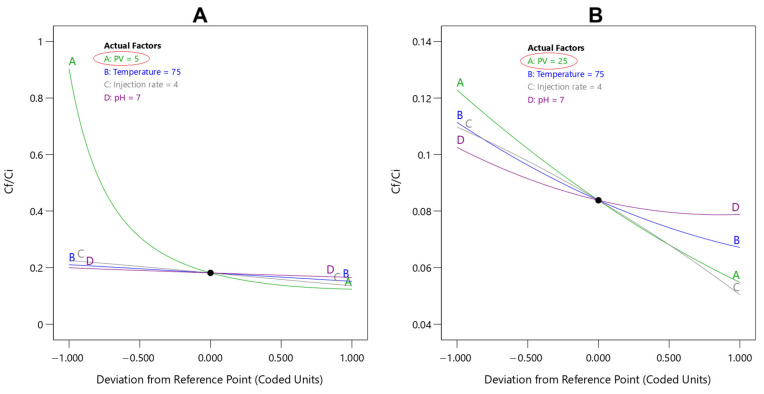
Sensitivity analysis for C_f_/C_i_-PV010 (**A**) and C_f_/C_i_-PV1040 (**B**).

Sensitivity analysis was performed to evaluate how key operating parameters influence the C_f_/C_i_ ratio. The resulting sensitivity curves for the C_f_/C_i_-PV010 and C_f_/C_i_-PV1040 models are presented in Figure 9A and Figure 9B, respectively. Figure 9A corresponds to the C_f_/C_i_-PV010 model and illustrates how each variable affects C_f_/C_i_ at low to moderate PVs (0–10). Figure 9B shows the C_f_/C_i_-PV1040 model, highlighting parameter importance at moderate to high PVs (10–40). In each plot, only one parameter was varied across its full range, while all others were fixed at the reference point (coded-unit “0” of all parameters) within the design space: temperature = 75 °C, injection rate = 4 mL/min, and pH = 7. The reference PV for Figure 9A was 5, and for Figure 9B was 25.

Figure 9 demonstrates that increasing PV, temperature, injection rate, or pH generally reduces the C_f_/C_i_. In Figure 9A, the pronounced curvature of the PV line shows that PV has the strongest influence on C_f_/C_i_ at low PVs, while the relatively flat curves for temperature, injection rate, and pH indicate that these parameters have minimal impact in this range. In contrast, Figure 9B shows that at higher PVs (e.g., PV = 25), C_f_/C_i_ becomes sensitive to nearly all parameters. Both PV and injection rate exhibit strong effects, whereas pH shows the least influence, with limited ability to alter C_f_/C_i_. These findings indicate that at high PVs, not only PV but also injection rate and temperature play critical roles in process optimization and in identifying conditions that maximize C_f_/C_i_.

It should be noted that at low at low PV values, the effect of PV on C_f_/C_i_ is much stronger because a large portion of the adsorbed PPCA still remains on the rock surface, so even small increases in PV cause significant desorption and sharp changes in effluent concentration. As PV increases, most accessible adsorption sites become depleted and the polymer layer becomes thinner, so additional injected PV produces smaller incremental changes in C_f_/C_i_ [22,34,67]. However, the higher-PV region is more important from a practical and mechanistic standpoint because it represents the long-term behavior of the treatment, the stability of the remaining polymer film, and the residual inhibitor availability under harsh reservoir conditions [24,51,68]. In the following sections, we will discuss the significance of this high-PV regime in more detail.

### 3.6. Parameter Effects

As discussed, the higher-PV region is more important from a practical and mechanistic standpoint. Therefore, the goal of this section is the investigation of main operating parameters on C_f_/C_i_ at relatively high PVs from 10 to 40.

**Figure 10:**
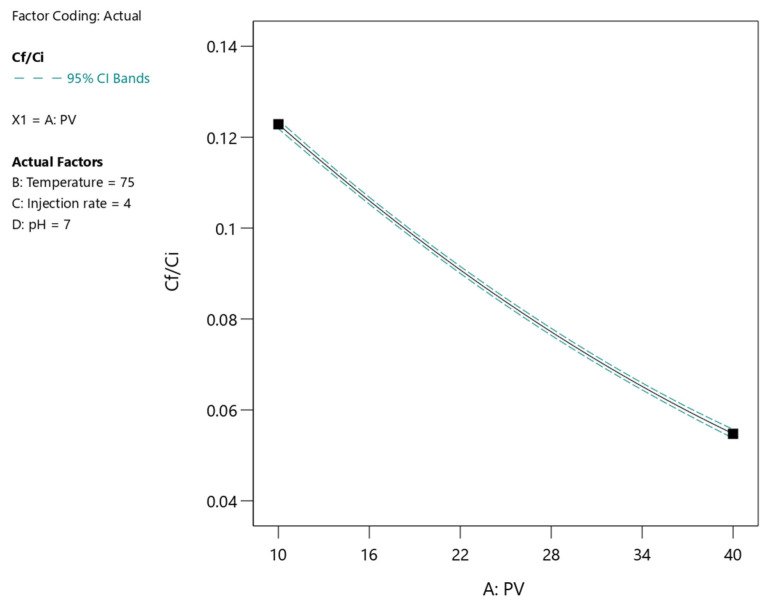
Effect of injected pore volume on predicted C_f_/C_i_ during PPCA desorption.

Figure 10 presents a one-factor response plot from the RSM model predicting the desorption ratio C_f_/C_i_ of the PPCA scale inhibitor during core-flooding. The x-axis represents pore volumes injected, varying from 10 to 40, while temperature (75 °C), pH (7), and injection rate (4 mL/min) are held constant. The downward-sloping curve shows that C_f_/C_i_ decreases steadily as PV increases, indicating progressive desorption and washout of the inhibitor from the rock surface [23,33]. At lower PV (about 10), the C_f_/C_i_ is relatively high (0.12) because a larger fraction of inhibitor remains available in the effluent. As more PVs are injected, adsorption sites become depleted and the retained inhibitor is gradually displaced, reducing the effluent concentration to 0.06 by 40 PV.

**Figure 11:**
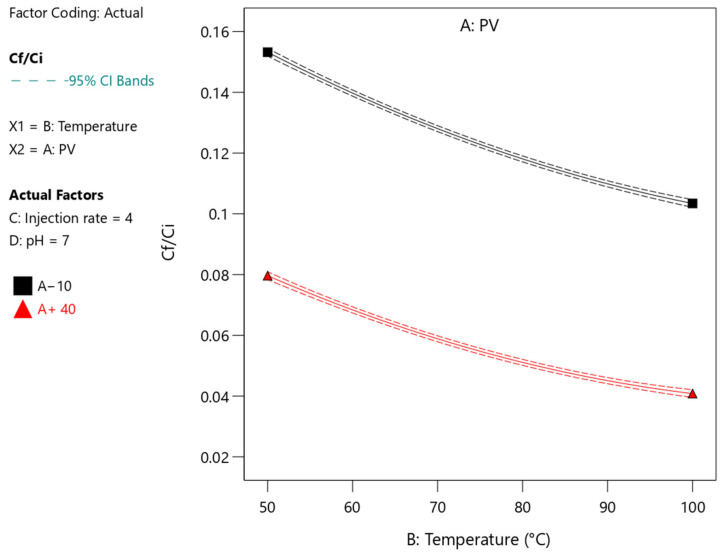
Effect of temperature on predicted C_f_/C_i_ for PPCA desorption at two pore volumes.

Figure 11 shows the response for temperature-dependent desorption of PPCA scale inhibitor, evaluated at two pore volumes (10 and 40) while keeping pH of 7 and injection rate of 4 mL/min constant. In both cases, C_f_/C_i_ decreases progressively with increasing temperature (50–100 °C), but the magnitude differs between the two PVs. At 10 PV, C_f_/C_i_ is higher overall because the polymer has not yet undergone extensive washout; adsorption sites still retain polymer chains, and desorption is dominated by early-stage displacement. At 40 PV, lower C_f_/C_i_ values reflect deeper polymer depletion and reduced surface coverage. Scientific behavior is consistent with polymer desorption thermodynamics. Increasing temperature weakens hydrogen bonding and other intermolecular interactions between PPCA chains and mineral surfaces, enhancing desorption [11,17,52]. Higher thermal energy also increases chain mobility, reduces solution viscosity, and promotes faster mass transfer, all contributing to a decline in C_f_/C_i_ [69].

**Figure 12:**
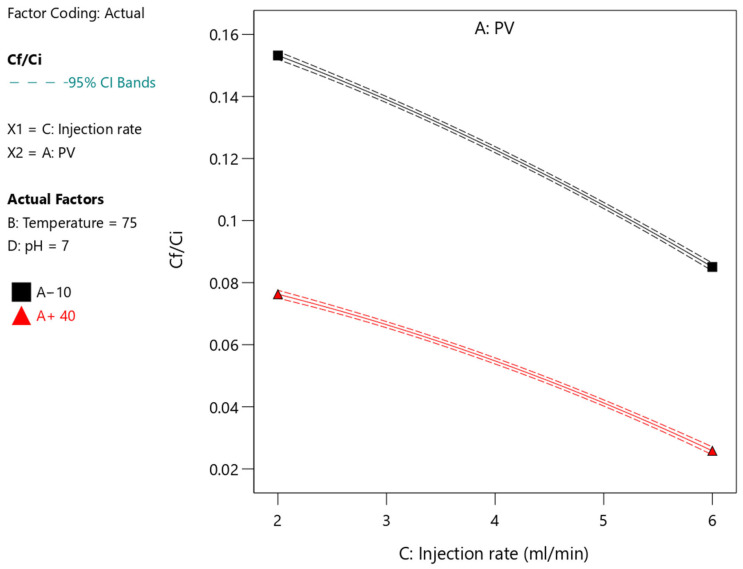
Effect of Injection Rate on Predicted Cf/Ci for PPCA Desorption at Two Pore Volumes.

Figure 12 illustrates the effect of injection rate (2–6 mL/min) on the desorption ratio C_f_/C_i_ of the PPCA polymer inhibitor at 75 °C and pH = 7, evaluated at PV = 10 and PV = 40. In both cases, C_f_/C_i_ decreases as the injection rate increases, reflecting faster removal of polymer from the core. At 10 PV, C_f_/C_i_ values are higher because the inhibitor is still relatively abundant on the pore surfaces, and desorption is governed by early-stage polymer release. At 40 PV, C_f_/C_i_ is significantly lower, indicating that the polymer has already undergone substantial depletion. The observed trends align with polymer transport and hydrodynamic principles. Higher injection rates increase linear velocity, reduce polymer residence time, and intensify shear forces within pore channels [70,71,72]. These effects weaken polymer-rock interactions, disrupt adsorbed polymer layers, and promote quicker displacement of PPCA chains. Additionally, elevated flow rates enhance mass-transfer gradients, accelerating desorption. Thus, increased injection rate leads to more efficient washout, especially when the system has already been exposed to high PV [24,73].

**Figure 13:**
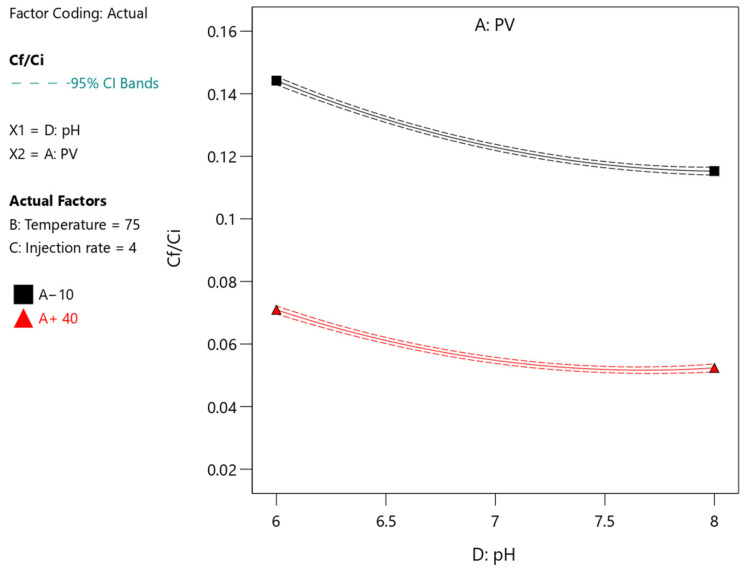
Effect of pH on Predicted C_f_/C_i_ for PPCA Desorption at Two Pore Volumes.

Figure 13 shows the effect of pH (6–8) on the desorption ratio C_f_/C_i_ of the PPCA scale inhibitor at 75 °C, 4 mL/min injection rate, and two pore volumes (PV = 10 and PV = 40). In both cases, C_f_/C_i_ decreases as pH increases, indicating enhanced desorption under more alkaline conditions. At PV = 10, C_f_/C_i_ decreases from about 0.145 at pH 6 to roughly 0.115 at pH 8. At PV = 40, C_f_/C_i_ falls from around 0.075 at pH 6 to approximately 0.055 at pH 8. The lower overall values at 40 PV reflect greater polymer depletion caused by prolonged flushing. This behavior is consistent with polymer chemistry and surface-interaction mechanisms. PPCA polymers contain acidic functional groups whose ionization increases with rising pH [52]. Higher ionization enhances electrostatic repulsion between polymer chains and between polymer and mineral surfaces, weakening adsorption strength. Increased charge density also improves polymer solubility and mobility, promoting desorption and lowering C_f_/C_i_ [51,74,75,76]. Thus, alkaline conditions favor faster inhibitor release, especially after extensive exposure (40 PV).

It should be noted that ionic strength and brine composition, particularly the concentration of divalent cations such as Ca^2+^ and Mg^2+^, can significantly influence the desorption behavior of polymeric scale inhibitors such as PPCA. Under higher salinity conditions, especially in carbonate reservoirs containing concentrated formation brines, increased ionic strength can compress the electrical double layer surrounding both the polymer chains and the mineral surface, thereby modifying electrostatic interactions. In addition, elevated concentrations of divalent cations may promote stronger complexation between PPCA functional groups and metal ions, enhancing inhibitor retention and potentially shifting the desorption plateau toward higher pore volumes, resulting in a longer inhibitor return period. Conversely, competitive ion exchange and charge screening effects may also alter polymer conformation and mobility, affecting release kinetics. Although brine salinity was kept constant in the present work to isolate the effects of PV, temperature, injection rate, and pH, the influence of ionic strength remains an important field-scale parameter and should be investigated in future studies for more comprehensive reservoir-specific squeeze design.

The observed desorption behavior of PPCA in the present study appears to be governed predominantly by surface adsorption/desorption mechanisms, rather than by precipitation/dissolution controlled solely through the solubility of a Ca-PPCA complex. This interpretation is supported by several observations. First, the inhibitor concentration used during the adsorption stage (40 ppm) was relatively low compared with concentrations typically associated with bulk Ca-PPCA precipitation reported in previous studies, reducing the likelihood of extensive precipitation within the porous medium. Second, the smooth and continuous decline in C_f_/C_i_ with increasing pore volume, without abrupt release peaks or secondary concentration maxima, suggests a gradual desorption process from surface-bound polymer layers rather than dissolution of discrete precipitated phases. Third, the strong sensitivity of the return profile to pH, temperature, and injection rate further indicates that the dominant mechanism is controlled by reversible interfacial interactions, including electrostatic attraction, hydrogen bonding, and surface complexation between PPCA functional groups and carbonate mineral sites. Nevertheless, localized formation of limited Ca-PPCA surface complexes at active calcium-rich sites on calcite and dolomite cannot be completely excluded and may contribute to stronger retention at early stages of desorption. Therefore, the overall return behavior is best described as adsorption-dominated desorption with possible minor contribution from surface-associated Ca-PPCA complexation, rather than a classical precipitation-squeeze dissolution mechanism.

**Figure 14:**
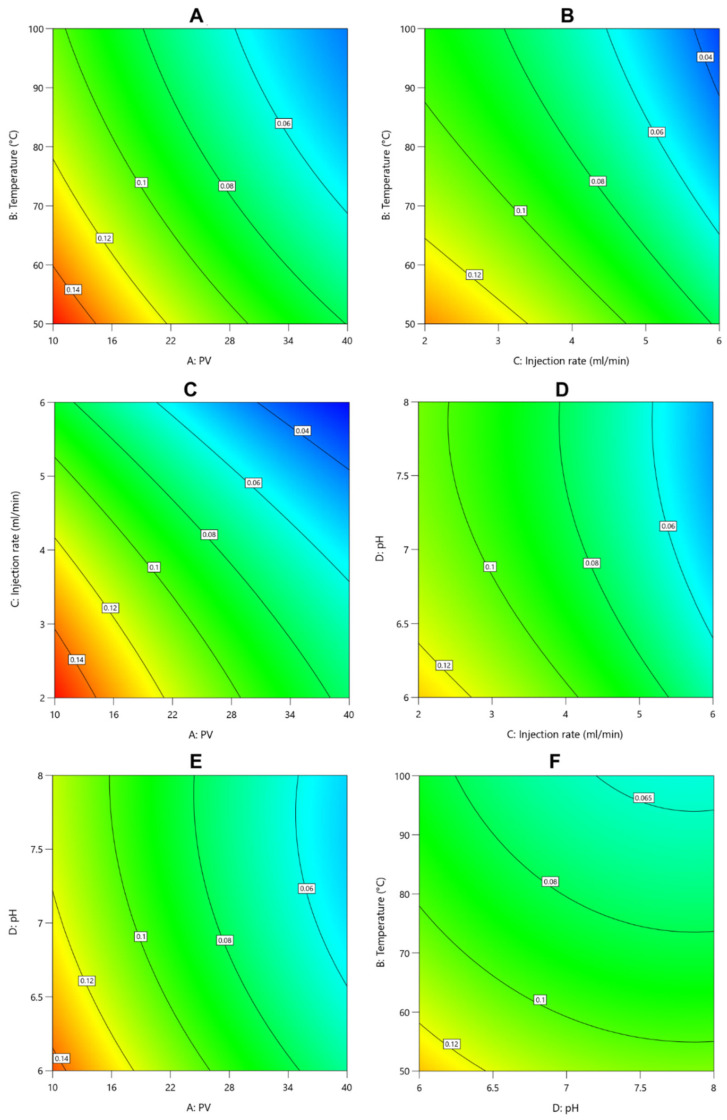
Contour Plots Illustrating Combined Parameter Influences on Predicted Cf/Ci. (**A**) the influence of temperature and PV on the Cf/Ci; (**B**) the influence of temperature and injection rate on the Cf/Ci; (**C**) the influence of injection rate and PV on the Cf/Ci; (**D**) the influence of pH and injection rate on the Cf/Ci; (**E**) the influence of pH and PV on the Cf/Ci; (**F**) the influence of temperature and pH on the Cf/Ci.

**Figure 15:**
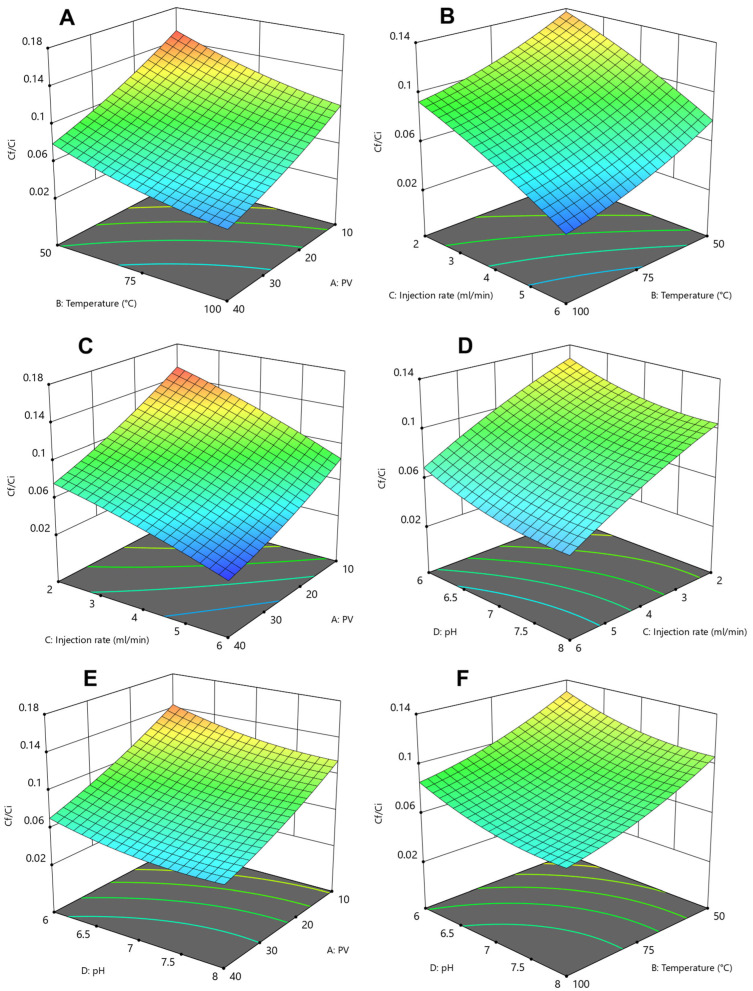
3-D response surfaces interaction effects of operational parameters on Cf/Ci. (**A**) the influence of temperature and PV on the Cf/Ci; (**B**) the influence of temperature and injection rate on the Cf/Ci; (**C**) the influence of injection rate and PV on the Cf/Ci; (**D**) the influence of pH and injection rate on the Cf/Ci; (**E**) the influence of pH and PV on the Cf/Ci; (**F**) the influence of temperature and pH on the Cf/Ci.

Figure 14 and Figure 15 present the contour and their corresponding 3-D response surfaces plots describing the combined influence of PV, temperature, injection rate, and pH on the desorption ratio C_f_/C_i_ of PPCA during core flooding. Each subplot (A–F) examines the interaction between two variables while holding the remaining two constant. Together, the figures visualize how simultaneous parameter changes modify inhibitor release behavior. Across all plots, C_f_/C_i_ decreases with increasing PV, temperature, injection rate, or pH, but the rate and magnitude depend on the interaction between variables. For example, in plots A and C, C_f_/C_i_ decreases from about 0.17 at low PV (10) and low temperature or injection rate, down to 0.05–0.06 at PV 40 combined with high temperature or high flow rate. This reflects the compounded effect of extended flushing (high PV) and enhanced desorption kinetics driven by thermal activation or hydrodynamic shear [52,77]. In plots B and D, increasing temperature (50–100 °C) together with higher injection rate (2–6 mL/min) produces stronger declines in C_f_/C_i_, reaching values as low as 0.03, due to reduced solution viscosity, accelerated molecular motion, and weakened polymer-surface interactions. Higher temperatures also reduce the stability of adsorbed PPCA layers, promoting displacement by faster flow [78]. For pH interactions (plots E and F), C_f_/C_i_ decreases from around 0.14 to 0.05 as pH increases from 6 to 8 combined with higher PV or temperature. Alkaline conditions increase PPCA ionization, enhancing electrostatic repulsion and weakening adsorption strength. Temperature amplifies this by increasing chain mobility, yielding synergistic desorption.

### 3.7. Optimization

The primary aim of the optimization process is to identify the optimal operating conditions, including PV, temperature, injection rate, and pH, that achieve the most favorable C_f_/C_i_ ratio under high PV and harsh reservoir simulated conditions. Since high PV represents extended polymer exposure, the optimization focuses on finding conditions that maximize inhibitor recovery efficiency, ensuring that desorption remains effective even when the system is subjected to elevated temperature and high flow rates. Process optimization was conducted using the RSM **C_f_/C_i_-PV1040 model** under high-PVs of 30–40 as presented in Table 8. The primary objective (Goal I) was to determine the optimal conditions under high PVs at a moderate temperature (60 °C), however, the second objective related to the worst-case scenario. In both cases, the pH is fixed at 7, reflecting real reservoir conditions where formation water typically shows near-neutral pH.

**Figure 16:**
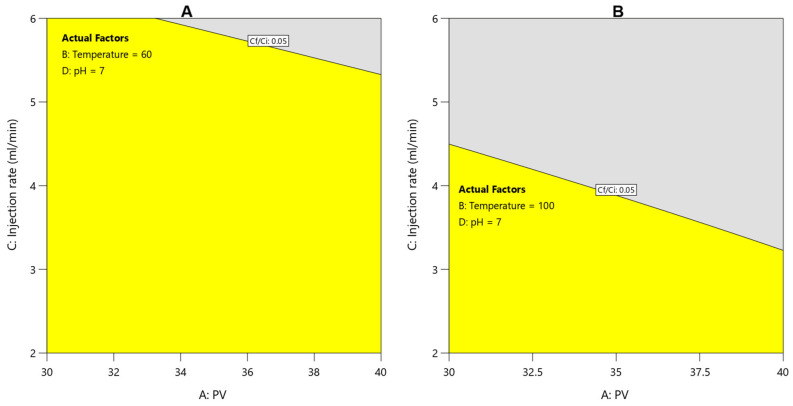
Optimization results showing conditions required to maintain the PPCA threshold at 60 °C (**A**) and 100 °C (**B**).

The optimization results for the mentioned objectives are presented in Table 8 and also illustrated in Figure 16. Figure 16A,B present the optimized operational regions required to maintain an effluent-to-injected concentration ratio (C_f_/C_i_) of 0.05, which represents the practical minimum concentration needed for effective field-scale scale inhibition. In these plots, the yellow region corresponds to C_f_/C_i_ ≥ 0.05, indicating operational conditions where inhibitor return remains above the practical threshold, ensuring sufficient protection against scale deposition. The grey region represents C_f_/C_i_ < 0.05, where inhibitor return is inadequate for sustained scale control. At 60 °C (Figure 16A), the yellow (effective) region is relatively broad, indicating that under moderate temperature, the PPCA inhibitor maintains adequate concentration across a wide range of PVs and injection rates. However, at 100 °C (Figure 16B), the yellow region becomes significantly smaller. The boundary for C_f_/C_i_ = 0.05 shifts downward much faster with increasing PV. Only limited combinations (at injection rates of less than 3.5–4.5 mL/min) remain in the effective region. Beyond these conditions, the system transitions into the grey zone, where C_f_/C_i_ falls below the effective inhibition threshold. At this high temperature, polymer desorption accelerates sharply. Thermal energy increases PPCA chain mobility, weakens adsorption forces, and enhances the mass-transfer-driven transport of polymer into the flowing brine. Even though pH remains near-neutral, the harsh thermal conditions dominate desorption behavior. Higher injection rates exacerbate this effect by increasing hydrodynamic shear and convective removal of inhibitor from pore surfaces.

It should be emphasized that the optimization presented in this study is intended as a laboratory-scale operational framework rather than a complete field squeeze-treatment design. The developed RSM model provides optimized injection conditions (PV, temperature, injection rate, and pH) required to maintain the inhibitor return concentration above the selected operational threshold (C_f_/C_i_ = 0.05), thereby establishing a scientifically justified basis for preliminary squeeze design and treatment screening. However, translation of these laboratory-derived results to field-scale applications requires incorporation of reservoir-specific parameters such as reservoir thickness, radial flow geometry, formation heterogeneity, wellbore conditions, produced water rate, and target squeeze lifetime.

In practical field applications, additional design variables—including inhibitor dosage, total squeeze volume, overflush design, and shut-in time—must be determined through reservoir simulation and field-specific scale-management workflows. These parameters are strongly dependent on formation mineralogy, adsorption capacity, near-wellbore transport behavior, and operational production constraints. Therefore, while the present work provides the fundamental desorption kinetics and optimized operational windows necessary for squeeze-treatment design, a full-scale predictive treatment design and long-term release model requires coupling the developed laboratory model with reservoir-scale transport simulators. Such integration is currently under development by the authors and will be presented in a future study focused specifically on field-scale squeeze-treatment design and lifetime prediction.

Therefore, the current optimization addresses laboratory-scale squeeze-performance optimization, while field implementation requires additional reservoir-specific engineering design.

### 3.8. Core Permeability Preservation During Calcium Sulfate Scale Formation

To further evaluate the scale inhibition performance of PPCA under porous-media conditions, additional permeability damage experiments were conducted by monitoring the normalized permeability ratio (K_d_/K_i_) as a function of injected pore volume during calcium sulfate scaling. The experiments were performed at a constant pH = 7 and injection rate of 4 mL/min, under both blank conditions (without inhibitor) and in the presence of 40 ppm PPCA at 50, 75, and 100 °C.

**Figure 17:**
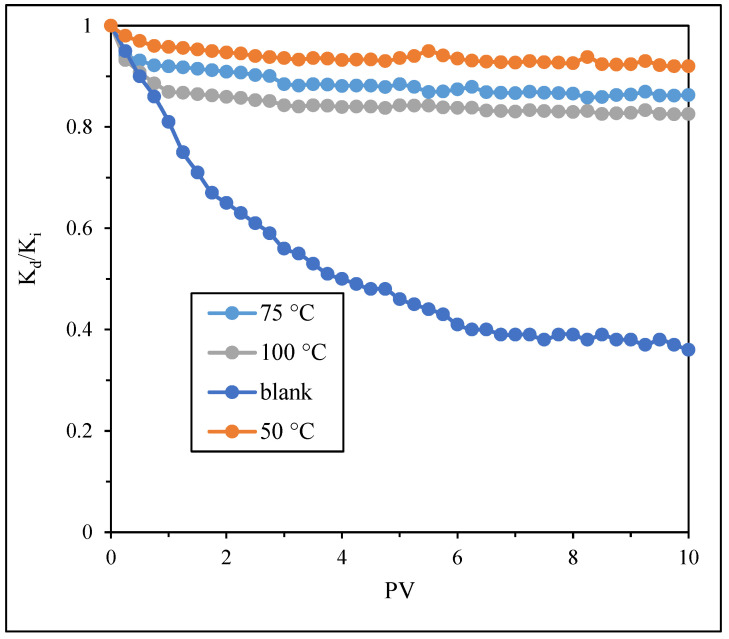
Effect of PPCA on normalized permeability (K_d_/K_i_) during calcium sulfate scaling at different temperatures.

As shown in Figure 17, severe permeability decline occurred under blank conditions, where K_d_/K_i_ decreased continuously from 1 to approximately 0.36 after 10 PV, indicating substantial pore plugging and nearly 64% permeability loss due to uncontrolled calcium sulfate deposition. In contrast, the presence of 40 ppm PPCA significantly preserved core permeability at all temperatures, confirming effective scale suppression inside the porous medium. At 50 °C, permeability remained relatively stable, with K_d_/K_i_ = 0.92 after 10 PV, corresponding to only 8% permeability damage, demonstrating excellent scale protection. At 75 °C, the final K_d_/K_i_ was approximately 0.863, indicating about 13.7% permeability reduction, while at 100 °C, K_d_/K_i_ decreased to around 0.826, corresponding to approximately 17.4% damage.

The slight reduction in permeability preservation at elevated temperatures is attributed to increased calcium sulfate nucleation and crystal growth kinetics, which partially reduces inhibitor effectiveness. Nevertheless, even at 100 °C, PPCA maintained more than 82% of the initial permeability, confirming strong thermal stability and effective inhibition under harsh reservoir conditions. These results further validate that the selected 40 ppm concentration (MIC) not only prevents bulk scale formation, as shown in static and dynamic tests, but also effectively protects formation permeability, which is a critical requirement for successful field-scale squeeze treatment design.

To improve the practical applicability of the present work, an optimization table of operational parameters (Table 9) has been added to the manuscript, summarizing the recommended ranges of pore volume, temperature, injection rate, and pH required to maintain effective inhibitor return performance under the studied conditions. These optimized operating windows provide a preliminary guideline for PPCA squeeze treatment design in carbonate reservoirs and may assist in selecting field-relevant injection conditions to prolong squeeze lifetime and improve scale control efficiency. From a practical perspective, maintaining moderate injection rates, controlling near-neutral pH conditions, and minimizing unnecessary thermal exposure were identified as favorable strategies for enhancing inhibitor retention and achieving more stable long-term release behavior. However, it should be emphasized that the present study primarily focuses on experimental evaluation and statistical modeling of desorption behavior, while full-scale field design, including dosage optimization, squeeze volume design, shut-in time recommendations, and long-term slow-release prediction equations—requires additional reservoir-specific modeling and field validation. These aspects are currently under investigation and will be addressed in a future dedicated study currently under preparation.

### 3.9. Adsorption-Desorption Mechanism and Interfacial Interaction of PPCA on Carbonate Rock Surfaces

The adsorption-desorption behavior of PPCA on carbonate rock surfaces is governed by a complex combination of surface chemistry, polymer conformation, electrostatic interactions, and flow-driven mass transfer phenomena. Because PPCA is a multifunctional anionic polymer containing both phosphino/phosphonate and carboxylate functional groups, its retention mechanism cannot be described as a simple monolayer adsorption process; rather, it involves multiple simultaneous interfacial interactions between polymer chains, dissolved ions, and mineral surface sites.

At the mineral surface, the dominant carbonate phases (calcite and dolomite) provide active Ca^2+^-rich adsorption sites capable of interacting strongly with negatively charged PPCA functional groups. The primary retention mechanism is believed to involve surface complexation, in which phosphonate and carboxylate groups form coordination bonds with exposed calcium atoms on the carbonate surface. Among these groups, phosphonate moieties generally exhibit stronger binding affinity due to their higher charge density and stronger metal-chelation capability, while carboxylate groups contribute additional secondary anchoring points and improve polymer spreading across the mineral interface. This leads to a multi-point attachment mechanism, where one polymer chain can simultaneously bind to several active sites, substantially increasing adsorption stability.

In addition to chemical complexation, electrostatic interactions strongly influence adsorption. At lower pH, partial protonation of PPCA functional groups reduces intramolecular charge repulsion, allowing the polymer chain to adopt a more compact conformation and approach the carbonate surface more easily, thereby enhancing adsorption. Conversely, at higher pH, increased deprotonation increases negative charge density along the polymer backbone, promoting chain expansion due to intramolecular repulsion and increasing electrostatic repulsion from negatively charged mineral sites, which weakens adsorption and favors desorption. This explains the experimentally observed decrease in C_f_/C_i_ at higher pH.

Polymer configuration within the pore space also plays a critical role. Once adsorbed, PPCA may form train-loop-tail structures, a classical polymer adsorption model, where “train” segments are directly attached to the mineral surface, while “loops” and “tails” extend into the aqueous phase. During desorption, loosely bound loop and tail segments are removed first, causing the initial rapid decline in return concentration. More strongly attached train segments desorb more slowly, explaining the gradual long-term tailing behavior observed at higher pore volumes. This multi-stage desorption behavior confirms that retention is not uniform but distributed across adsorption sites of different energies.

Temperature further modifies this mechanism by altering both polymer mobility and interfacial bond stability. Higher temperature increases molecular motion and decreases solution viscosity, which enhances transport and accelerates desorption. Simultaneously, thermal agitation weakens hydrogen bonding and reduces the lifetime of surface complexes, further facilitating polymer release. Similarly, increasing injection rate reduces polymer residence time and increases hydrodynamic shear, which can disrupt weakly adsorbed polymer layers and accelerate convective removal.

Overall, the adsorption-desorption mechanism of PPCA in carbonate media is best interpreted as a dynamic balance between multi-site surface complexation, polymer conformational rearrangement, electrostatic forces, and hydrodynamic displacement. This mechanistic understanding explains the strong sensitivity of PPCA return profiles to pore volume, temperature, injection rate, and pH, and provides an important scientific basis for optimizing squeeze treatment performance in carbonate reservoirs.

## 4. Conclusions

This study presented a comprehensive experimental and modeling investigation of the desorption behavior of the polymeric scale inhibitor PPCA in carbonate core samples. Dynamic core flooding experiments were conducted under varying operational conditions, including pore volume, temperature, injection rate, and pH, to simulate realistic reservoir environments. Using Response Surface Methodology, two predictive models for low (0–10 PV) and high (10–40 PV) pore volume ranges were developed. The developed RSM models showed excellent agreement with experimental data, with high R^2^ values (>99%), confirming their reliability for predicting inhibitor behavior. The division of the modeling domain into low and high pore volume regions provided improved accuracy in capturing both early-stage and long-term desorption behavior.

The results demonstrated that among the investigated parameters, pore volume and injection rate were identified as the most influential factors controlling inhibitor desorption. Increasing pore volume led to continuous reduction in C_f_/C_i_, confirming that extended flushing enhances inhibitor removal due to mass transfer-controlled mechanisms. Similarly, higher injection rates reduced residence time and increased hydrodynamic forces, accelerating polymer displacement. Temperature also played a critical role, as increasing temperature from 50 to 100 °C significantly enhanced desorption by weakening polymer-rock interactions and increasing molecular mobility. In contrast, pH exhibited a relatively smaller but still noticeable effect, where higher pH values promoted desorption due to increased ionization and electrostatic repulsion of the polymer chains.

In addition to desorption and core-flood modeling results, supplementary experimental investigations were performed to strengthen the scale inhibition assessment of PPCA. Static jar tests confirmed high inhibition efficiency (more than 90%) at and above 40 ppm under different temperature conditions, while identifying 40 ppm as MIC for effective calcium sulfate scale control. Dynamic tube blocking experiments further demonstrated the superior performance of PPCA, where stable pressure behavior and minimal pressure build-up (less than 15 kPa) were observed at MIC conditions, whereas severe blockage (higher 400 kPa) occurred at lower concentrations. Moreover, permeability reduction analysis (K_d_/K_i_) confirmed that PPCA significantly mitigates scale-induced formation damage compared to blank systems, highlighting its protective role in carbonate porous media. The integration of these results with RSM-based desorption modeling provides a more complete evaluation of PPCA, covering both scale inhibition efficiency and long-term squeeze performance.

Furthermore, optimization analysis revealed that maintaining a minimum effective concentration (C_f_/C_i_ ≥ 0.05) is feasible over a broad range of conditions at moderate temperatures (60 °C), while at higher temperatures (100 °C), the operational window becomes significantly restricted.

## Figures and Tables

**Table 1:** Ionic composition of formation and injection waters.

Ion Type	Ionic Concentration (ppm)	
	Formation Water	Injection Water
**Sodium**	37,258	9845
**Calcium**	6000	266
**Potassium**	876	121
**Magnesium**	613	1021
**Chloride**	82,341	19,867
**Sulfate**	96	2043
**TDS (ppm)**	127,184	33,163

**Table 2:** Mineralogical composition of carbonate core samples used in this study.

Mineral Phase	Composition	Measurement Uncertainty (wt%)
**Calcite**	68	2
**Dolomite**	27	2
**Quartz**	3	2
**Clay**	2	1
**Total**	100	-

**Table 3:** The experimental levels of the analyzed parameters.

Parameter	Symbol	Unit	Experimental Levels (Coded and Actual)		
			−1	0	+1
PV	A	°C mL/min	from 0 to 40		
Temperature	B		50	75	100
Injection rate	C		2	4	6
pH	D		6	7	8

**Table 4:** Developed RSM-models for prediction of C_f_/C_i_ in terms of coded factors.

	Developed RSM-Models	
Name	Formula	Application
**C_f_/C_i_-PV010**	**(C_f_/C_i_)^−0.5^** = 0.3684 + 0.1926 A − 0.0026 B − 0.0662 C + 0.0687 D + 0.0008 AB + 0.0179 AC + 0.0076 AD − 0.0178 A^2^ + 4.1729 × 10^−5^ B^2^ + 0.0153907 C^2^	**0 < PV ≤ 10**
**C_f_/C_i_-PV1040**	**C_f_/C_i_** = 0.0838 − 0.0340 A − 0.0221 B − 0.0296 C − 0.0118 D + 0.0027 AB + 0.0044 AC + 0.0026 AD + 0.0050 A^2^ + 0.0055 B^2^ − 0.0037 C^2^ + 0.0068 D^2^	**10 ≤ PV ≤ 40**

**Table 5:** ANOVA result of the C_f_/C_i_-PV010 model.

Source	Sum of Squares	df	Mean Square	F-Value	*p*-Value	
**Model** **A-PV** **B-Temperature** **C-Injection rate** **D-pH** **AB** **AC** **AD** **A^2^** **B^2^** **C^2^** **Residual** **Cor Total**	38.56 30.21 1.13 2.74 0.3573 0.0918 0.3157 0.0144 2.08 0.0131 0.0728 0.2573 38.82	10 1 1 1 1 1 1 1 1 1 1 101 111	3.86 30.21 1.13 2.74 0.3573 0.0918 0.3157 0.0144 2.08 0.0131 0.0728 0.0025	1513.73 11,857.37 442.36 1076.05 140.25 36.05 123.94 5.65 815.97 5.13 28.56	<0.0001 <0.0001 <0.0001 <0.0001 <0.0001 <0.0001 <0.0001 0.0193 <0.0001 0.0257 <0.0001	**significant**

**Table 6:** ANOVA result of the C_f_/C_i_-PV1040 model.

Source	Sum of Squares	df	Mean Square	F-Value	*p*-Value	
**Model** **A-PV** **B-Temperature** **C-Injection rate** **D-pH** **AB** **AC** **AD** **A^2^** **B^2^** **C^2^** **D^2^** **Residual** **Cor Total**	0.1004 0.0490 0.0157 0.0281 0.0045 0.0001 0.0002 0.0001 0.0003 0.0003 0.0001 0.0005 0.0002 0.1006	11 1 1 1 1 1 1 1 1 1 1 1 100 111	0.0091 0.0490 0.0157 0.0281 0.0045 0.0001 0.0002 0.0001 0.0003 0.0003 0.0001 0.0005 2.075 × 10^−6^	4396.03 23,627.99 7568.70 13,560.05 2174.18 43.75 114.79 39.13 152.62 153.35 71.31 242.47	<0.0001 <0.0001 <0.0001 <0.0001 <0.0001 <0.0001 <0.0001 <0.0001 <0.0001 <0.0001 <0.0001 <0.0001	**significant**

**Table 7:** Fit statistics for the developed models.

No.	Fit Statistics		Model	
			C_f_/C_i_-PV010	C_f_/C_i_-PV1040
**1**	Coefficient of determination	R^2^	0.9934	0.9979
**2**	Adjusted coefficient of determination	Adj-R^2^	0.9927	0.9977
**3**	Predicted coefficient of determination	Pred-R^2^	0.9915	0.9972
**4**	Adequate precision	AP	151.3442	270.1669
**5**	Coefficient of variation	C.V. %	2.40	1.63

**Table 8:** The optimization goals and corresponding results using RSM C_f_/C_i_-PV1040.

Parameters	Unit	Goals		Optimization Results	
		I	II	I	II
**Pore volume (PV)**	-	High PVs (30–40)	High PVs (30–40)	30–40	30–40
**Temperature**	°C	in range	highest value (100)	60	100
**Injection rate**	mL/min	in range	in range	<5.5–6	<3.5–4.5
**pH**	-	in range	in range	7	7
**C_f_/C_i_**	-	**>0.05**	**>0.05**	**>0.05**	**>0.05**

**Table 9:** Optimal conditions derived modeling for a worst-case scenario in the presence of PPCA for scale inhibition.

PV	Temperature (°C)	pH	Injection Rate (mL/min)	PPCA Concentration (ppm)	K_d_/K_i_ (Experimental)
**10**	**100**	**8**	**2**	**40**	**0.805**

## Data Availability

The data used is provided in the article and can be used.

## Appendix A

**Table A1:** DOE-RSM experimental design and results.

Run	Parameters				Response
	PV	Temperature	Injection Rate	pH	Cf/Ci
		°C	mL/min		
**1**	0	50	4	7	1
**2**	0	75	4	7	1
**3**	0	100	4	7	1
**4**	0	75	2	7	1
**5**	0	75	6	7	1
**6**	0	75	4	6	1
**7**	0	75	4	8	1
**8**	0.5	50	4	7	0.875
**9**	0.5	75	4	7	0.805
**10**	0.5	100	4	7	0.745
**11**	0.5	75	2	7	0.886
**12**	0.5	75	6	7	0.732
**13**	0.5	75	4	6	0.844
**14**	0.5	75	4	8	0.766
**15**	1	50	4	7	0.717
**16**	1	75	4	7	0.622
**17**	1	100	4	7	0.506
**18**	1	75	2	7	0.742
**19**	1	75	6	7	0.488
**20**	1	75	4	6	0.685
**21**	1	75	4	8	0.532
**22**	1.5	50	4	7	0.592
**23**	1.5	75	4	7	0.511
**24**	1.5	100	4	7	0.405
**25**	1.5	75	2	7	0.634
**26**	1.5	75	6	7	0.389
**27**	1.5	75	4	6	0.561
**28**	1.5	75	4	8	0.436
**29**	2	50	4	7	0.511
**30**	2	75	4	7	0.445
**31**	2	100	4	7	0.347
**32**	2	75	2	7	0.547
**33**	2	75	6	7	0.336
**34**	2	75	4	6	0.48
**35**	2	75	4	8	0.371
**36**	2.5	50	4	7	0.422
**37**	2.5	75	4	7	0.375
**38**	2.5	100	4	7	0.298
**39**	2.5	75	2	7	0.457
**40**	2.5	75	6	7	0.261
**41**	2.5	75	4	6	0.395
**42**	2.5	75	4	8	0.321
**43**	3	50	4	7	0.355
**44**	3	75	4	7	0.315
**45**	3	100	4	7	0.257
**46**	3	75	2	7	0.369
**47**	3	75	6	7	0.224
**48**	3	75	4	6	0.337
**49**	3	75	4	8	0.281
**50**	3.5	50	4	7	0.311
**51**	3.5	75	4	7	0.274
**52**	3.5	100	4	7	0.237
**53**	3.5	75	2	7	0.334
**54**	3.5	75	6	7	0.201
**55**	3.5	75	4	6	0.296
**56**	3.5	75	4	8	0.25
**57**	4	50	4	7	0.281
**58**	4	75	4	7	0.234
**59**	4	100	4	7	0.19
**60**	4	75	2	7	0.296
**61**	4	75	6	7	0.176
**62**	4	75	4	6	0.264
**63**	4	75	4	8	0.195
**64**	4.5	50	4	7	0.244
**65**	4.5	75	4	7	0.207
**66**	4.5	100	4	7	0.165
**67**	4.5	75	2	7	0.259
**68**	4.5	75	6	7	0.153
**69**	4.5	75	4	6	0.225
**70**	4.5	75	4	8	0.179
**71**	5	50	4	7	0.216
**72**	5	75	4	7	0.185
**73**	5	100	4	7	0.149
**74**	5	75	2	7	0.23
**75**	5	75	6	7	0.137
**76**	5	75	4	6	0.201
**77**	5	75	4	8	0.162
**78**	5.5	50	4	7	0.193
**79**	5.5	75	4	7	0.164
**80**	5.5	100	4	7	0.14
**81**	5.5	75	2	7	0.209
**82**	5.5	75	6	7	0.127
**83**	5.5	75	4	6	0.179
**84**	5.5	75	4	8	0.153
**85**	6	50	4	7	0.182
**86**	6	75	4	7	0.155
**87**	6	100	4	7	0.133
**88**	6	75	2	7	0.197
**89**	6	75	6	7	0.114
**90**	6	75	4	6	0.168
**91**	6	75	4	8	0.143
**92**	7	50	4	7	0.165
**93**	7	75	4	7	0.148
**94**	7	100	4	7	0.123
**95**	7	75	2	7	0.185
**96**	7	75	6	7	0.106
**97**	7	75	4	6	0.158
**98**	7	75	4	8	0.137
**99**	8	50	4	7	0.159
**100**	8	75	4	7	0.137
**101**	8	100	4	7	0.113
**102**	8	75	2	7	0.172
**103**	8	75	6	7	0.097
**104**	8	75	4	6	0.154
**105**	8	75	4	8	0.129
**106**	9	50	4	7	0.154
**107**	9	75	4	7	0.13
**108**	9	100	4	7	0.106
**109**	9	75	2	7	0.163
**110**	9	75	6	7	0.093
**111**	9	75	4	6	0.146
**112**	9	75	4	8	0.122
**113**	10	50	4	7	0.149
**114**	10	75	4	7	0.124
**115**	10	100	4	7	0.1
**116**	10	75	2	7	0.155
**117**	10	75	6	7	0.085
**118**	10	75	4	6	0.147
**119**	10	75	4	8	0.116
**120**	12	50	4	7	0.143
**121**	12	75	4	7	0.119
**122**	12	100	4	7	0.097
**123**	12	75	2	7	0.147
**124**	12	75	6	7	0.081
**125**	12	75	4	6	0.137
**126**	12	75	4	8	0.11
**127**	14	50	4	7	0.139
**128**	14	75	4	7	0.112
**129**	14	100	4	7	0.09
**130**	14	75	2	7	0.142
**131**	14	75	6	7	0.075
**132**	14	75	4	6	0.133
**133**	14	75	4	8	0.104
**134**	16	50	4	7	0.134
**135**	16	75	4	7	0.107
**136**	16	100	4	7	0.087
**137**	16	75	2	7	0.136
**138**	16	75	6	7	0.072
**139**	16	75	4	6	0.125
**140**	16	75	4	8	0.099
**141**	18	50	4	7	0.13
**142**	18	75	4	7	0.101
**143**	18	100	4	7	0.082
**144**	18	75	2	7	0.129
**145**	18	75	6	7	0.068
**146**	18	75	4	6	0.124
**147**	18	75	4	8	0.095
**148**	20	50	4	7	0.125
**149**	20	75	4	7	0.097
**150**	20	100	4	7	0.078
**151**	20	75	2	7	0.124
**152**	20	75	6	7	0.062
**153**	20	75	4	6	0.113
**154**	20	75	4	8	0.091
**155**	22	50	4	7	0.121
**156**	22	75	4	7	0.092
**157**	22	100	4	7	0.073
**158**	22	75	2	7	0.118
**159**	22	75	6	7	0.057
**160**	22	75	4	6	0.11
**161**	22	75	4	8	0.085
**162**	24	50	4	7	0.115
**163**	24	75	4	7	0.086
**164**	24	100	4	7	0.069
**165**	24	75	2	7	0.112
**166**	24	75	6	7	0.052
**167**	24	75	4	6	0.104
**168**	24	75	4	8	0.081
**169**	26	50	4	7	0.11
**170**	26	75	4	7	0.0801
**171**	26	100	4	7	0.066
**172**	26	75	2	7	0.108
**173**	26	75	6	7	0.048
**174**	26	75	4	6	0.1
**175**	26	75	4	8	0.079
**176**	28	50	4	7	0.105
**177**	28	75	4	7	0.076
**178**	28	100	4	7	0.061
**179**	28	75	2	7	0.101
**180**	28	75	6	7	0.043
**181**	28	75	4	6	0.095
**182**	28	75	4	8	0.074
**183**	30	50	4	7	0.1
**184**	30	75	4	7	0.072
**185**	30	100	4	7	0.058
**186**	30	75	2	7	0.096
**187**	30	75	6	7	0.04
**188**	30	75	4	6	0.091
**189**	30	75	4	8	0.068
**190**	32	50	4	7	0.096
**191**	32	75	4	7	0.068
**192**	32	100	4	7	0.055
**193**	32	75	2	7	0.092
**194**	32	75	6	7	0.037
**195**	32	75	4	6	0.087
**196**	32	75	4	8	0.063
**197**	34	50	4	7	0.091
**198**	34	75	4	7	0.063
**199**	34	100	4	7	0.05
**200**	34	75	2	7	0.088
**201**	34	75	6	7	0.033
**202**	34	75	4	6	0.082
**203**	34	75	4	8	0.061
**204**	36	50	4	7	0.088
**205**	36	75	4	7	0.061
**206**	36	100	4	7	0.048
**207**	36	75	2	7	0.082
**208**	36	75	6	7	0.031
**209**	36	75	4	6	0.077
**210**	36	75	4	8	0.058
**211**	38	50	4	7	0.085
**212**	38	75	4	7	0.058
**213**	38	100	4	7	0.046
**214**	38	75	2	7	0.08
**215**	38	75	6	7	0.028
**216**	38	75	4	6	0.075
**217**	38	75	4	8	0.055
**218**	40	50	4	7	0.082
**219**	40	75	4	7	0.055
**220**	40	100	4	7	0.044
**221**	40	75	2	7	0.076
**222**	40	75	6	7	0.025
**223**	40	75	4	6	0.071
**224**	40	75	4	8	0.052
